# Diffusible signal factor (DSF) quorum sensing signal and structurally related molecules enhance the antimicrobial efficacy of antibiotics against some bacterial pathogens

**DOI:** 10.1186/1471-2180-14-51

**Published:** 2014-02-27

**Authors:** Yinyue Deng, Amy Lim, Jasmine Lee, Shaohua Chen, Shuwen An, Yi-Hu Dong, Lian-Hui Zhang

**Affiliations:** 1 Institute of Molecular and Cell Biology, Proteos, 61 Biopolis Drive, Singapore 138673, Singapore; 2 Guangdong Province Key Laboratory of Microbial Signals and Disease Control, College of Natural Resources and Environment, South China Agricultural University, Guangzhou, People’s Republic of China

## Abstract

**Background:**

Extensive use of antibiotics has fostered the emergence of superbugs that are resistant to multidrugs, which becomes a great healthcare and public concern. Previous studies showed that quorum sensing signal DSF (diffusible signal factor) not only modulates bacterial antibiotic resistance through intraspecies signaling, but also affects bacterial antibiotic tolerance through interspecies communication. These findings motivate us to exploit the possibility of using DSF and its structurally related molecules as adjuvants to influence antibiotic susceptibility of bacterial pathogens.

**Results:**

In this study, we have demonstrated that DSF signal and its structurally related molecules could be used to induce bacterial antibiotic susceptibility. Exogenous addition of DSF signal (*cis*-11-methyl-2-dodecenoic acid) and its structural analogues could significantly increase the antibiotic susceptibility of *Bacillus cereus,* possibly through reducing drug-resistant activity, biofilm formation and bacterial fitness. The synergistic effect of DSF and its structurally related molecules with antibiotics on *B. cereus* is dosage-dependent. Combination of DSF with gentamicin showed an obviously synergistic effect on *B. cereus* pathogenicity in an *in vitro* model. We also found that DSF could increase the antibiotic susceptibility of other bacterial species, including *Bacillus thuringiensis*, *Staphylococcus aureus*, *Mycobacterium smegmatis*, *Neisseria subflava* and *Pseudomonas aeruginosa*.

**Conclusion:**

The results indicate a promising potential of using DSF and its structurally related molecules as novel adjuvants to conventional antibiotics for treatment of infectious diseases caused by bacterial pathogens.

## Background

Antibiotics, which act by either killing or stopping microbial growth, have been used extensively in the control and prevention of infectious diseases. However, this live-or-die selection pressure has inevitably fostered the emergence of superbugs which are resistant to a range of conventional antibiotics. Infections associated with antibiotic-resistant pathogens are becoming more and more common in clinical and nosocomial settings [1,2], which become severe healthcare and public concerns. In addition, antibiotics are commonly associated with a range of adverse effects [3]. For instance, treatment using aminoglycoside antibiotics, such as gentamicin and kanamycin, can cause serious side effects, including balance difficulty, hearing loss, and nephrotoxicity [4,5]. Reduction and limitation of antibiotic usage is therefore of critical importance in clinical treatment of microbial infections.

Combination antibiotics containing more than one antimicrobial agent are designed to either improve efficacy through synergistic action of the agents, or overcome the bacterial resistance. This method has been effectively used for treatment of tuberculosis, leprosy, malaria, HIV, infections associated with cystic fibrosis, and infective endocarditis [6-9]. Currently, antibiotic combinations are frequently used to provide empirical treatment for serious infections. However, given the facts that effective antibiotic combinations are still limited and superbugs are emerging rapidly, it is essential to continue to search for effective antibiotic combinations and other novel approaches to control infectious diseases. Recently, using nonantibiotic molecules to enhance the antibacterial efficacy of antibiotics offers a new kind of opportunity to practice a previously untapped expanse of clinical treatments. A few combinations of nonantibiotics with antibiotics showed increased activity against bacterial pathogens *in vitro* and *in vivo*[8,10-12].

The diffusible signal factor (DSF), which was originally found in *Xanthomonas campestris* pv *campestris* (*Xcc*), represents a new family of widely conserved quorum sensing (QS) signals in many Gram-negative bacterial species. It has been well-established that DSF-family signals play important roles in regulation of various biological functions such as biofilm formation, motility, virulence and antibiotic resistance [13-21]. In addition to their key roles in intraspecies signaling, the importance of DSF-family signals in interspecies and inter-kingdom communication has also been recognized [18,22]. It was reported that DSF signals from *Burkholderia cenocepacia* and *Stenotrophomonas maltophilia* modulate the virulence, antibiotic resistance and persistence of *Pseudomonas aeruginosa* in the cystic fibrosis airway [23,24]. Furthermore, it was found that an DSF-family signal produced by *P. aeruginosa* not only disperses its own biofilm formation but could also induce dispersion of biofilms of *Escherichia coli*, *Klebsiella pneumoniae*, *Proteus mirabilis*, *Streptococcus pyogenes*, *Bacillus subtilis*, *Staphylococcus aureus*, and the yeast *Candida albicans*[25]. Moreover, DSF-family signals showed a high level of potency in interference of the morphology transition of *C. albicans*[14,17,22], which is a critical feature associated with the virulence of this pathogen.

Given the fact that biofilm formation is related to antibiotic resistance [26], together with the role of DSF-family signals in regulation of bacterial biofilm formation and antibiotic resistance, we speculate that DSF-family signals may have a role in modulation of bacterial antibiotic susceptibility. In this study, we report that in the presence of DSF signal and its derivatives, some of which were identified as bacterial quorum sensing (QS) signals [13,14,18,22], the minimum inhibitory concentrations (MIC) of a few antibiotics against the bacterial pathogens were significantly reduced. Furthermore, we showed that supplementation of DSF signal could substantially enhance the antimicrobial activity of gentamicin and reduce the cytotoxicity of *B. cereus* in an *in vitro* infection model. Our findings suggest the promising potentials of DSF and its structurally related molecules as putative antibiotic adjuvants for the control of bacterial infections.

## Results

### DSF and its structurally related molecules increase the antibiotic susceptibility of *B. cereus*

**Figure 1:**
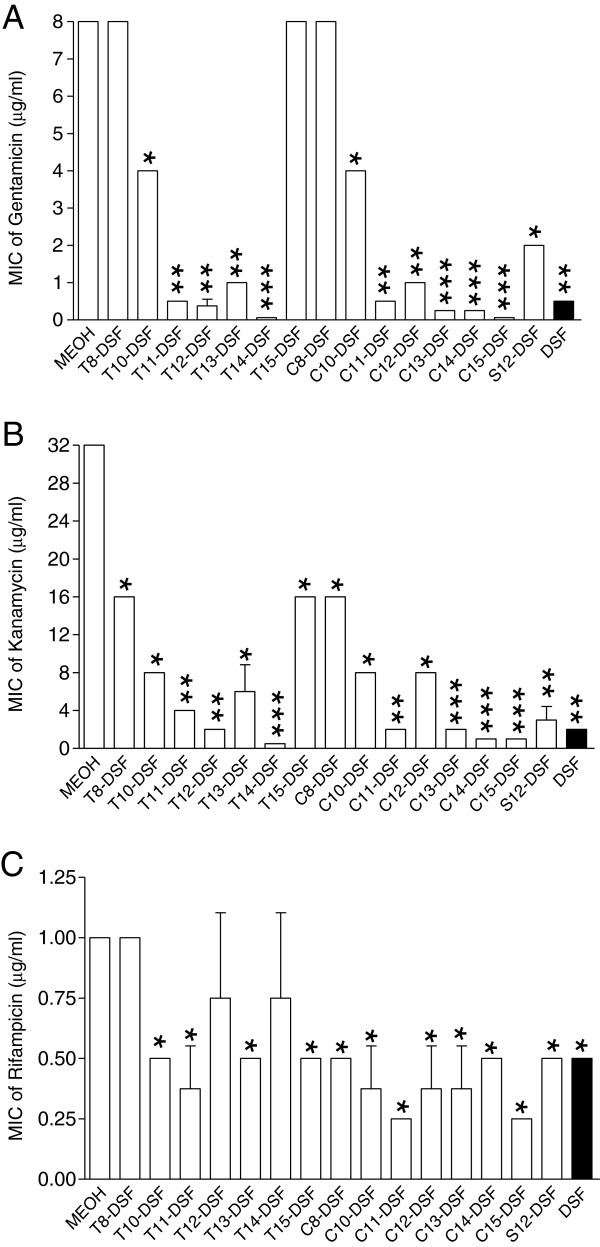
**Synergistic activity of DSF and its structurally related molecules (50 μM) with gentamicin (A), kanamycin (B), and rifampicin (C) against *B. cereus*.** For each antibiotic, a series 2-fold dilution was prepared for determination of MIC with or without DSF or related molecule. Data shown are means of two replicates and error bars indicate the standard deviations. The differences between the samples with addition of 50 μM DSF or related molecule and control are statistically significant with *p < 0.05, **p < 0.01, ***p < 0.001, as determined by using the Student t test.

*Bacillus* is a genus of Gram-positive, rod-shaped bacteria. They are ubiquitous in nature, and consisting of both free-living and pathogenic species. *Bacillus* bacteria produce oval endospores to endure a wide range of extreme environmental conditions, while keeping the capacity to return to vegetative growth [27]. This remarkable characteristics of the endospore-vegetative cell transition of *Bacillus* pathogens allows them to be utilized as biological weapons [28,29]. Interestingly, our preliminary results showed that this morphological transition between the vegetative cell and endospore of *Bacillus* species could be stopped by exogenous addition of DSF-family signals (Deng, unpublished data). This finding, together with the previous observations that DSF signals are involved in regulation of bacterial biofilm formation, antibiotic tolerance and fungal morphological transition [15,22-24], we speculated that DSF-family signals may affect the bacterial antibiotic sensitivity of *Bacillus* cells. To test this hypothesis, we firstly chose *B. cereus*, which is a common human pathogen and causes foodborne illness such as nausea, vomiting and diarrhea [30], to assay the antibiotic susceptibility in the presence of DSF signal or its derivatives (Table 1). The result showed that except for T8-DSF, T15-DSF and C8-DSF, all the other DSF-family signals and structurally related molecules displayed significantly synergistic effects with gentamicin (Figure 1A), which is an aminoglycoside and inhibits bacterial protein synthesis mainly through binding with the 30S ribosomal subunit. In particular, addition of T14-DSF or C15-DSF decreased the MIC of gentamicin against *B. cereus* from 8.0 μg/ml to 0.0625 μg/ml, which represents a 128-fold difference (Figure 1A). Similarly, addition of DSF and related molecules to *B. cereus* culture also enhanced the bacterial susceptibility to kanamycin from 2- to 64-fold with T14-DSF showing the strongest synergistic activity (Figure 1B). Interestingly, kanamycin is also an aminoglycoside that interacts with the 30S subunit of prokaryotic ribosomes and inhibits protein synthesis. Compared to the strong synergistic effect on gentamicin and kanamycin, DSF and related molecules showed only moderate effects on rifampicin, addition of these molecules increased the antibiotic sensitivity of *B. cereus* up to 4-fold (Figure 1C). Different from gentamicin and kanamycin, rifampicin inhibits the DNA-dependent RNA polymerase in bacterial cells, thus preventing gene transcription to generate RNA molecules and subsequent translation to synthesize proteins.

**Table 1:** Chemical structure of DSF signal and its derivatives used in this study

**Compound**	**Configuration**	**Structure**	**References**
T8-DSF	*trans*		14
T10-DSF	*trans*		14
T11-DSF	*trans*		14
T12-DSF	*trans*		14
T13-DSF	*trans*		14
T14-DSF	*trans*		14
T15-DSF	*trans*		14
C8-DSF	*cis*		14
C10-DSF	*cis*		14
C11-DSF	*cis*		14
C12-DSF	*cis*		22
DSF	*cis*		14
C13-DSF	*cis*		This study
C14-DSF	*cis*		14
C15-DSF	*cis*		14
S12-DSF	NT		This study

### The synergistic activity of DSF and its structurally related molecules with antibiotics on *B. cereus* is dosage-dependent

**Figure 2:**
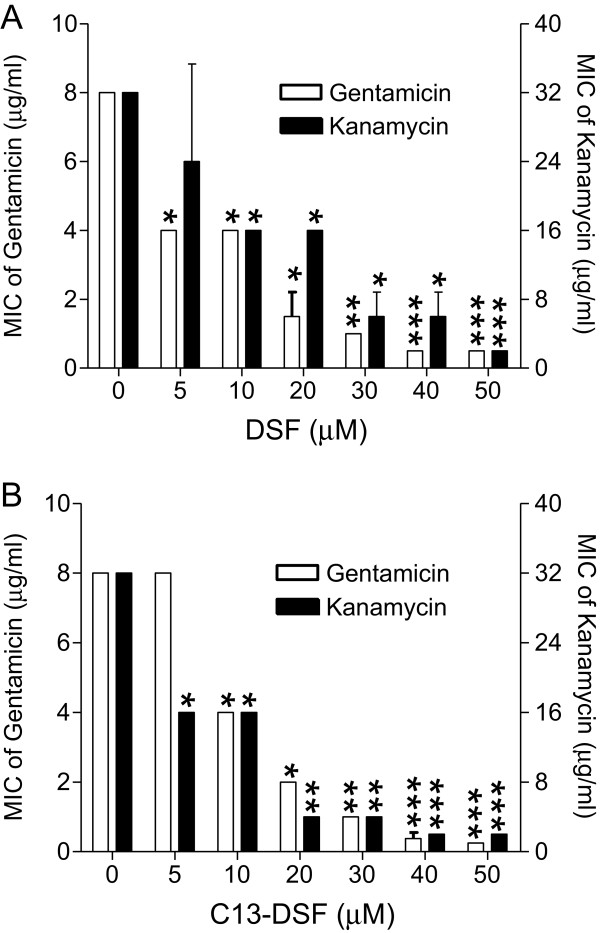
**Synergistic activity of different concentrations of DSF (A) and C13-DSF (B) with gentamicin and kanamycin on *B. cereus*.** Data shown are means of two replicates and error bars indicate the standard deviations. The differences between the samples with addition of DSF or C13-DSF and control are statistically significant with *p < 0.05, **p < 0.01, ***p < 0.001, as determined by using the Student t test.

To determine whether the synergistic activity of DSF with antibiotics is related to its dosages, DSF was supplemented to the growth medium at various final concentrations, and MICs of gentamicin and kanamycin against *B. cereus* were tested. The results showed that activity of DSF signal on *B. cereus* sensitivity to gentamicin and kanamycin was dependent on the final concentration of the signal molecule (Figure 2A). Addition of DSF at a final concentration from 5 – 50 μM increased the antibiotic susceptibility of *B. cereus* to gentamicin by 2- to 16-fold, respectively (Figure 2A). Similarly, as shown in Figure 2A, combination of different final concentrations of DSF signal with kanamycin increased the synergistic activity by 1.3- to 16-fold.

To test the dosage-dependent synergistic activity of other DSF related molecules, we selected C13-DSF, which was prepared abundantly in our laboratory, as a representative molecule for further analysis. As shown in Figure 2B, the effects of C13-DSF on *B. cereus* sensitivity to gentamicin and kanamycin were also dosage-dependent. Addition of C13-DSF at a final concentration from 10 μM to 50 μM increased the gentamicin susceptibility of *B. cereus* by 2- to 32-fold, and similarly, increased the bacterial kanamycin susceptibility by about 2- to 16-fold (Figure 2B).

### Combination of DSF signal with gentamicin synergistically decreases *B. cereus* pathogenicity in *in vitro* assays

**Figure 3:**
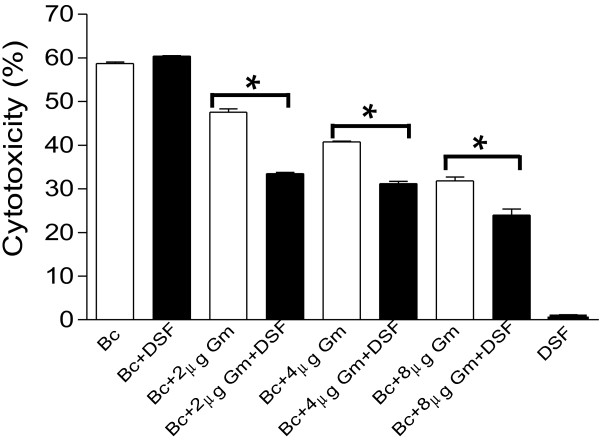
**The synergistic effect of DSF signal (50 μM) with gentamicin on the virulence of *B. cereus *in an *in vitro *model.** Cytotoxicity was assayed by monitoring LDH release by the HeLa cells infected with a MOI of about 1000. Data shown are means of three replicates and error bars indicate the standard deviations. The differences between the samples with DSF and without DSF are statistically significant with *p < 0.05, as determined by using the Student t test.

We then continued to investigate the possibility of using DSF signal as antibiotics adjuvant for the therapy of infectious diseases caused by bacterial pathogens. HeLa cells were used as the *in vitro* model to test the synergistic activity of DSF signal with antibiotics against *B. cereus*. Results showed that exogenous addition of gentamycin significantly decreased the cytotoxicity of *B. cereus* to HeLa cell. For 2.5 h inoculation, the cytotoxicity of *B. cereus* was reduced by 11.15%, 17.95%, and 26.9%% with supplementation of 2, 4, and 8 μg/ml gentamycin, respectively (Figure 3). In contrast, combination of 50 μM DSF signal with gentamycin led to more decreased cytotoxicity of *B. cereus* to HeLa cell than addition of the antibiotic alone. As shown in Figure 3, the cytotoxicity of *B. cereus* to HeLa cells was reduced by 26.9%, 29.15% and 36.4 with treatment of 2, 4, and 8 μg/ml gentamycin in combination with 50 μM DSF, respectively. As a control, we found that DSF signal showed no cytotoxicity to HeLa cells and didn’t affect the *B. cereus* virulence (Figure 3). These results not only further confirm the synergistic effect of DSF signal with antibiotics on *B. cereus*, but also highlight the potentials of using DSF and its structurally related molecules as adjuvants to antibiotics for treatment of infectious diseases caused by bacterial pathogens.

### DSF signal interferes with the drug-resistant activity, biofilm formation and persistence of *B. cereus*

**Figure 4:**
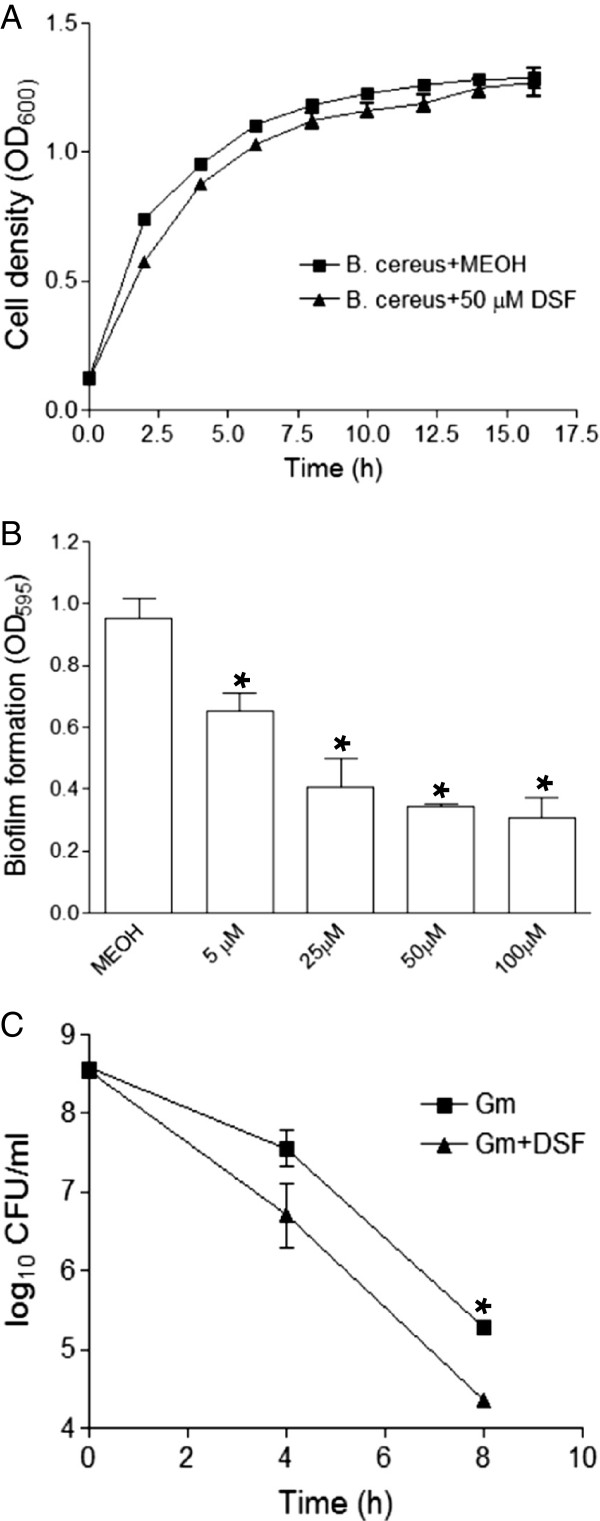
**Influences of exogenous addition of DSF signal on the bacterial growth rate (A) biofilm formation (B), and persistence (C) of *B. cereus*.** For measurement of growth rate, the bacterial cells were grown in the absence or presence of 50 μM DSF; while for test of persistence, the bacterial cells were treated with10 μg/ml gentamicin (Gm) in the absence or presence of 50 μM DSF signal. For biofilm formation assays, DSF signal was added at different final concentrations as indicated. Data shown are means of three replicates and error bars indicate the standard deviations. The differences between the samples with DSF and without DSF are statistically significant with *p < 0.05, as determined by using the Student t test.

To elucidate the mode of action of DSF-family signals on *B. cereus*, we firstly analyzed the global gene expression patterns of *B. cereus* 10987 in the presence of DSF signal using microarray assay. It was revealed that addition of DSF signal significantly decreased the transcripts levels of the genes encoding a series of drug efflux systems and drug resistance proteinsof *B. cereus* (Additional file 1: Figure S1, Additional file 1: Table S1), which may likely reduce the antibiotic-resistant activity. We then tested the effect of DSF signal on *B. cereus* growth and biofilm formation. As shown in Figure 4, the growth rate of *B. cereus* was only slightly reduced with addition of 50 μM DSF signal, whereas the bacterial biofilm formation was substantially inhibited. Intriguingly, we also discovered that DSF signal remarkably reduced the persistence of *B. cereus* (Figure 4C). Addition of 50 μM DSF signal decreased the persistence rate of *B. cereus* by 5.5- and 8.7- fold after 4 h and 8 h incubation, respectively (Figure 4C). As bacterial biofilm and persisters are highly tolerant to different types of antibiotics, inhibition of biofilm formation and persistence may likely alter bacterial antibiotic susceptibility. In combination, our results suggest that DSF signal could exert multifaceted effect on *B. cereus*, such as reducing the drug-resistant activity, inhibiting biofilm formation and attenuating bacterial persistence, which might lead to altered bacterial susceptibility to antibiotics.

### The combination effect of DSF signal with antibiotics on other bacterial species

To study whether DSF could also influence the antibiotic susceptibility of other bacterial species, the signal was used to test the synergistic effect with antibiotics against a few bacterial species in our collection, including *Bacillus thuringiensis*, *Staphylococcus aureus*, *Mycobacterium smegmatis*, *Neisseria subflava*, and *Pseudomonas aeruginosa*. Among them, *B. thuringiensis* belongs to *B. cereus* group and has been used as a biopesticide for many years [31]. It is closely related to the other two member of *B. cereus* group, i.e., *B. anthracis* and *B. cereus*, which are important human pathogens to cause anthrax and foodborne illness, respectively [32]. *S. aureus* is frequently found in human respiratory tract and on the skin. It can cause a range of serious illnesses such as pneumonia, meningitis, osteomyelitis, endocarditis, toxic shock syndrome (TSS), bacteremia, and sepsis [33]. *M. smegmatis* is a useful model organism for research analysis of other Mycobacteria species, especially *M. tuberculosis*. It is generally considered to be a non-pathogenic bacterium, however, in rare cases it may also cause diseases [34]. *N. subflava* is a rare opportunistic pathogen and has been associated with endocarditis, bacteremia, meningitis, septic arthritis, endophthalmitis, and septicemia [35]. *P. aeruginosa* is a ubiquitous environmental organism that can infect animals, plants, and insects, and is a major source of opportunistic infections in immunocompromised patients and cystic fibrosis individuals [36].

As shown in Table 2, addition of DSF signal at a final concentration of 50 μM decreased the MICs of ampicillin, rifampicin, kanamycin, gentamicin, tetracycline, chloramphenicol, and trimethoprim against *B. thuringiensis* by 75%, 75%, 93.75%, 93.75%, 50%, 50%, and 75%, respectively. We then continued to test the synergistic effect of DSF signal with antibiotics against *S. aureus*. Inclusion of DSF signal at a final concentration of 50 μM caused reduction of the MICs of ampicillin, kanamycin and gentamicin by 50%, 50%, and 87.5%, respectively (Table 2). While for *M. smegmatis*, addition of DSF signal increased its susceptibility to kanamycin, gentamicin, chloramphenicol and trimethoprim by 75%, 50%, 50% and 50%, respectively (Table 2). For the synergistic effect of DSF signal with antibiotics against the Gram-negative bacterial pathogens, as shown in Table 2, it was found that addition of DSF only reduced the MICs of kanamycin and gentamicin against *N. subflava* and *P. aeruginosa* by 50%, respectively, but did not affect the MICs of other antibiotics against these two pathogens. Furthermore, we also studied the effect of DSF-family signals on the growth rate of these bacteria, as shown in Additional file 1: Figure S2, exogenous addition of DSF-family signals showed no influence on the growth of *P. aeruginosa*, but they slightly affected the growth of *B. thuringiensis*, *S. aureus* and *M. smegmatis*; and inhibited the growth of *N. subflava*, which may affect its synergistic effect with antibiotics on this particular pathogen.

**Table 2:** Synergistic activity of DSF signal (50 μM) with antibiotics against various bacterial species

		**MIC (μg/ml)**						
**Bacteria**		Gm*	**Km**	**Rm**	**Am**	**Tc**	**Cm**	**Tm**
*B. thuringiensis*	MEOH	4	32	1	1	4	4	512
	DSF	0.25	2	0.25	0.25	2	2	128
*S. aureus*	MEOH	0.125	2	0.0625	2	4	4	NA#
	DSF	0.016	1	0.0625	1	4	4	NA
*M. smegmatis*	MEOH	0.16	0.32	NA	256	0.16	6.4	0.64
	DSF	0.08	0.08	NA	256	0.16	3.2	0.32
*N. subflava*	MEOH	2	8	0.5	2	2	0.5	128
	DSF	1	4	0.5	2	2	0.5	128
*P. aeruginosa*	MEOH	1.28	128	NA	128	32	128	64
	DSF	0.64	64	NA	128	32	128	64

*Abbreviations: *Gm* gentamicin, *Km* kanamycin, *Rm* rifampicin, *Am* ampicillin, *Tc* tetracycline, *Cm* chloramphenicol, and *Tm* trimethoprim.

# NA means the bacterial species was not sensitive to the tested antibiotic.

## Discussion

Previous studies have established the significant roles of DSF-family signals in microbial ecology as well as in intraspecies signaling regulation [14,17,22-24,37]. It was reported that DSF signals could modulate various biological functions including virulence, biofilm formation, antibiotic resistance and persistence through interspecies communication [23,24,37]. Additionally, DSF-family signals were also found to play a role in inter-kingdom communication by inhibiting morphological transition of *C. albicans*[14,17,22]. The results from this study present a new role of DSF and its structurally related molecules, i.e., increasing the antibiotic susceptibility of some bacterial species (Figure 1, Table 2). Given that DSF at a final concentration of 5 μM, which appears to be a physiological relevant concentration [14,22], could substantially increase bacterial sensitivity to antibiotics (Figure 2A), it appears plausible that DSF-family signals may have a role in shaping local microbial ecology as they could reduce the competitive advantage of some community residents by down regulation of their antibiotic or toxin tolerance. Furthermore, our results also suggest that DSF and its structurally related molecules may be used as a new kind of antibiotic adjuvant for the treatment of infectious diseases caused by bacterial pathogens, subjecting to further evaluation of their toxicological and pharmacological properties.

DSF-family signals share a fatty acid carbon chain with variations in chain length, double-bond configuration, and side-chain [18]. Evidence is emerging that these structural features may contribute to their biological activity in intraspecies signalling and interspecies communication [14,17,37]. Our study showed that the synergistic activity of DSF and its structurally related molecules with antibiotics is influenced by their structural features. Each of these molecules has a distinct synergistic activity among which the disparity could be up to 128-fold (Figure 1A). As a general rule, our results showed that the unsaturated long chain DSF related molecules have better synergistic activity with antibiotics, especially the aminoglycoside antibiotics, than the short chain and saturated molecules. Meanwhile, the synergistic activity of DSF and related molecules may also seem to be affected by the mode of action of antibiotics as the synergistic activities of DSF and related molecules with aminoglycoside antibiotics such as gentamicin and kanamycin were much better than with other types of antibiotics (Figure 1, Table 2).

It was reported that BDSF signalling system positively regulates the antibiotic resistance of *B. cenocepacia*[21]. The same research group also found that addition of DSF signal to *P. aeruginosa* could increase the bacterial antibiotic tolerance to polymyxins [23]. Intriguingly, our results suggest an opposite effect of DSF and its structurally related molecules by increasing the bacterial antibiotic susceptibility (Figure 1, Table 2). The contradictory results may be due to the differences in the bacterial species or strains and the antibiotics used in studies, which is evident from our results (Table 2). It should also be noted that DSF-family signals were shown to play dual roles in regulation of biofilm formation as they positively control the biofilm development in some bacterial species, and they could also disperse the biofilms of other bacterial species [15,19,21,37].

Our results suggest that DSF and related molecules may influence the bacterial antibiotic susceptibility by multiple ways, including modulation of the biofilm formation, antibiotic resistant activity and bacterial persistence (Figure 4; Additional file 1: Table S1). In addition, we also examined the possibility of DSF and related molecules acting as biosurfactants to influence bacterial susceptibility to antibiotics by using rhamnolipid, which is a well characterized biosurfactants, as a control in MIC and growth analysis. We found that rhamnolipid could also increase the antibiotic susceptibility of *B. cereus* at the final concentration of 50 μM (data not shown), but it also inhibits bacterial growth at this concentration and its toxicity on *B. cereus* cells was at least 5-fold higher than DSF (Additional file 1: Figure S3), which complicates the comparison. With all considered, at this stage we could not rule out the possibility that DSF and related molecules may have biosurfactant property and this property may contribute to their synergistic effects with antibiotics. Furthermore, several lines of evidence from this study and previous reports seem to suggest that the signalling activity of DSF and its structurally related molecules may contribute to their ability in changing bacterial antibiotic susceptibility. Firstly, it was reported that BDSF signalling system positively controls the antibiotic resistance in *B. cenocepacia*, and addition of 50 μM DSF signal increased the antibiotic resistance of *P. aeruginosa* to polymyxins [21,23], indicating that DSF-family signals are possibly widely involved in regulation of bacterial antibiotic resistance. Secondly, different from rhamnolipid which has a strong hydrophilic head group glycosyl, DSF and related molecules only have a very weak hydrophilic activity, suggesting that they could not be good surfactants. This notion appears to be supported by the different inhibitory activity of DSF and rhamnolipid on the growth of *B. cereus* (Additional file 1: Figure S3). Thirdly, our findings showed that addition of 50 μM DSF signal showed no cytotoxicity to HeLa cells, didn’t affect the *B. cereus* virulence (Figure 3), but could significantly change the expression patterns of many genes in *B. cereus*, some of which are known to be associate with antibiotics resistance or tolerance (Additional file 1: Table S1). Fourthly, the synergistic activity of DSF is antibiotic specific. While DSF and its structurally related molecules have strong synergistic effect with gentamicin and kanamycin against *B. cereus*, they showed a moderate effect with rifampicin, or even no synergistic effect with other antibiotics such as ampicillin, tetracycline (Data not shown), which may not be solely explainable with biosurfactant properties. Fifthly, the synergistic effect of DSF with antibiotics is also bacterial species specific. We showed that DSF signal had a strong synergistic effect with gentamicin against *B. cereus*, *B. thuringiensis* and *S. aureus*, while it had only a moderate effect with gentamicin against *M. smegmatis*, *N. subflava* and *P. aeruginosa* (Figure 1, Table 2). In particular, DSF signal did not show any synergistic activity with any of the tested antibiotics, including gentamicin, kanamycin, rifampicin, ampicillin, tetracycline, chloramphenicol, and trimethoprim, against *Escherichia coli* (Data not shown). Finally, DSF and its structurally related molecules share a very similarly chemical structure, hydrophobic and hydrophilic properties, suggesting that they should have similar chemical properties. However, their synergistic activities were significantly different with disparity up to 128 folds (Figure 1A).

Taken together, the results from this study have established the role of DSF and its structurally related molecules in modulation of antibiotic susceptibility in some but not all bacterial pathogens. It is also clear that the synergistic activity with antibiotics is related to the structural features of DSF-related molecules and likely the chemical property or the mode of action of antibiotics. At least stage, it is not clear how DSF and its structurally related molecules could influence bacterial antibiotic sensitivity. Much work remains to be done to determine whether their functionality in modulating bacterial antibiotic sensitivity is related to their pure chemical properties such as biosurfactant or hydrophobic activities, or associated with their potential roles in interference of bacterial signalling and regulatory networks, or both. In this regard, DSF and its analogues may be served as a useful tool to probe the potential mechanisms governing bacterial sensitivity to antibiotics.

## Conclusions

In summary, we showed that DSF and its structurally related molecules could significantly increase bacterial susceptibility to antibiotics, especially gentamycin and kanamycin. Our data showed that the unsaturated long chain DSF related molecules have better synergistic activity with antibiotics, especially the aminoglycoside antibiotics, than the short chain and saturated molecules. This synergistic effect is generic on both Gram-positive and Gram-negative bacteria, but the tested Gram-positive bacteria appeared to be more sensitive to the activity of DSF and its structurally related molecules than the tested Gram-negative bacteria. The findings from this study suggest that DSF and its structurally related molecules may be used as antibiotic adjuvants, which could be useful for reducing the dose of antibiotics, hence minimizing the side effect caused by the antibiotics, and slowing down the development of antibiotic resistance.

## Methods

### Bacterial growth conditions and MIC assays

Bacterial strains used in this work are listed in Additional file 1: Table S2. Overnight cultures of bacteria were inoculated at an OD_600_ of 0.025 in LB broth supplemented with antibiotic in the absence and presence of DSF or its structural analogue (Table 1). One hundred microliters of inoculated culture were grown in each well at 28°C or 37°C as indicated with shaking at 200 rpm for 24 hours (Additional file 1: Table S2). MIC was defined as the lowest concentration of antibiotic in which bacterial growth in the well was not measureable by determination of the turbidity at 600 nm, and determined following the method from the Clinical and Laboratory Standards Institute (CLSI) [38].

### Bacterial growth analysis

Overnight bacterial cultures grown in LB broth were inoculated in the same medium to an OD_600_ of 0.025 in the absence and presence of DSF or its analogue at a final concentration of 50 μM. Three hundred microliters of inoculated culture were grown in each well at 28°C or 37°C as indicated in Additional file 1: Table S2 in a low intensity shaking model using the Bioscreen-C Automated Growth Curves Analysis System (OY Growth Curves AB Ltd., Finland).

### Biofilm formation assays

Biofilm formation was assayed using 96-well polypropylene microtitre dishes. Overnight bacterial cultures grown in LB broth were inoculated in the same medium to an OD_600_ of 0.01 in the absence and presence of DSF signal at different concentrations as indicated. One hundred microliters of inoculated culture were grown in each well at 37°C with shaking at 150 rpm for 18 h. The cultures were removed and 200 μl of 1% crystal violet (w/v) was added. Following staining at room temperature for 15 min, the dye was removed and the wells were rinsed three times with water. For quantification of the attached bacterial cells, the stained wells were decolorized with 200 μl of 95% ethanol. The quantity of crystal violet was determined by measuring the absorbance at 595 nm.

### Persistence assays

Persistence was measured by determining the number of cfu/mL after exposure to 10 μg/mL gentamicin. Overnight cultures were diluted 100-fold in 10 mL of fresh medium and incubated at 37°C at 250 rpm to an OD_600_ of 1.0. Cultures were incubated with shaking at 150 rpm at 37°C supplemented with gentamicin in the absence and presence of DSF signal at a final concentration of 50 μM. For determination of cfu, 1-mL aliquots were removed at the indicated time points and cells were serially diluted in fresh medium and plated on solid medium. Persisters were calculated after incubation at 37°C overnight.

### Cytotoxicity assays in HeLa cell model

The synergistic effect of DSF signal with antibiotic on the virulence of *B. cereus* was assayed by using HeLa cells. HeLa cells were seeded in 24-well tissue culture plates containing Dulbecco’s Modified Eagle Medium (DMEM) and allowed to grow at 37°C in CO_2_ for about 18 hours to obtain 80-90% monolayer confluency (5.0×10^5^ cells/well). Culture supernatants were removed and the monolayer was washed once with PBS buffer. Fresh bacterial cells cultured to an OD_600_ of 1.0 were diluted in DMEM with or without DSF at a final concentration of 50 μM, which were then added to the HeLa cell monolayers at a multiplicity of infection (MOI) about 1000, and gentamycin was added at different final concentrations as indicated. Cytotoxicity was determined by measuring the release of the cytosolic enzyme lactate dehydrogenase (LDH) into supernatants using the cytotoxicity detection kit (Roche).

## Competing interests

The authors declare that they have no competing interests.

## Authors’ contributions

Experiments were carried out by YD, AL, JL, SC, SA, YHD. Data analysis was finished by YD and LHZ. The study was designed by YD and LHZ, who also drafted the manuscript. All authors read and approved the final manuscript.

## Supplementary Material

Additional file 1: Figure S1 Real-time PCR analysis of DSF effect on transcriptional expression of selected genes in *B. cereus* 10987. **Table S1.** The genes with increased or decreased expression in *B. cereus* 10987 after treatment with 50 μM DSF. **Figure S2.** The bacterial growth rate in the presence and absence of 50 μM DSF or its analogue. **Figure S3.** Effect of DSF signal and rhamnolipid on the growth rate of *B. thuringiensis*. **Table S2.** Bacterial strains used in this study. Click here for file
