# Supplementary material for: Diffusible signal factor (DSF) quorum sensing signal and structurally related molecules enhance the antimicrobial efficacy of antibiotics against some bacterial pathogens
Source: BMC Microbiol. 2014 Feb 27;14:51. doi: 10.1186/1471-2180-14-51 (PMC3941561; doi:10.1186/1471-2180-14-51)
Supplement: Additional file 1: Figure S1 — Real-time PCR analysis of DSF effect on transcriptional expression of selected genes in B. cereus 10987. Table S1. The genes with increased or decreased expression in B. cereus 10987 after treatment with 50 μM DSF. Figure S2. The bacterial growth rate in the presence and absence of 50 μM DSF or its analogue. Figure S3. Effect of DSF signal and rhamnolipid on the growth rate of B. thuringiensis. Table S2. Bacterial strains used in this study. [file 1471-2180-14-51-S1.docx]

**Support Information**

Diffusible signal factor (DSF) quorum sensing signal and its structurally related moleculesenhance the antimicrobial efficacy of antibiotics against some bacterial pathogens

Yinyue Deng^1, 2^, Amy Lim^1^, Jasmine Lee^1^, Shaohua Chen^2^, Chao Wang^1^, Shuwen An^1^, Yi-Hu Dong^1^, and Lian-Hui Zhang^1, 2^*

*^1^Institute of Molecular and Cell Biology, Proteos, 61 Biopolis Drive, Singapore 138673, Singapore*

*^2^Guangdong Province Key Laboratory of Microbial Signals and Disease Control, College of Natural Resources and Environment, South China Agricultural University, Guangzhou, People's Republic of China*

**Experimental methods:**

**RNA extraction and Microarray analysis**

*B. cereus* 10987 was grown in LB medium in the absence and presence of 50 μM DSF till OD_600_ of 1.5. Total RNA was isolated using the RNeasy mini kit (Qiagen) according to the manufacturer’s instructions. The concentration and purity of RNA were determined by agarose gel electrophoresis and spectrometry. Microarray assay experiment was finished by the Genomax company under a commercial contact, which is briefly described as followed.

cDNA was synthesized from total RNA samples by using random primers (Invitrogen). SuperScript II (Invitrogen) and biotin-ddUTP was used to label the product according to the protocol from Affymetrix (Affymetrix). Target hybridization, washing and staining were performed according to the manufacture’s instructions. Genechip arrays were scanned with an Affymetrix probe array scanner. The microarray analysis for each bacterial strain was repeated for two times and the data were analyzed using a statistics software MAS-5.0 from Affymetrix.

**qRT-PCR analysis**

qRT-PCR quantifications were performed with a LightCycler® (Roche™) and QuantiFast SYBR Green (Qiagen™) according to the manufacturers’ instructions. The experiments were performed in triplicate, and the data were determined from two independent experiments. Expression levels were normalized to levels of the 16S RNA gene transcript in each experiment.


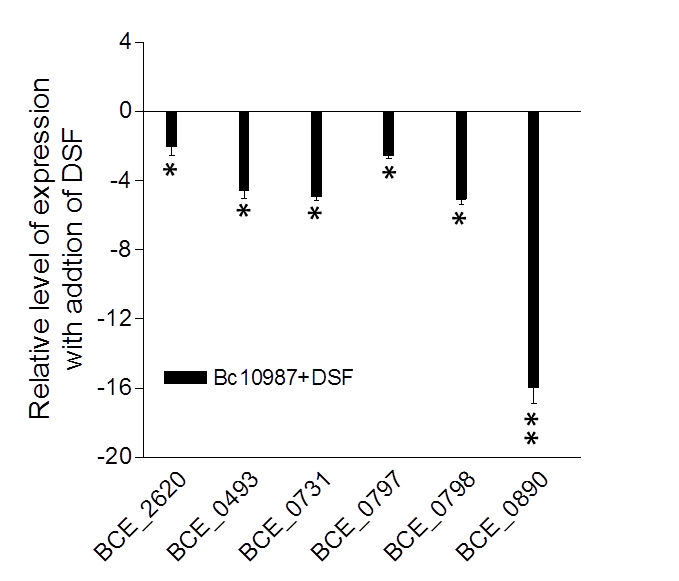


**Figure S1.** Real-time PCR analysis of DSF effect on transcriptional expression of selected genes in *B. cereus* 10987. Data shown are means of two replicates and error bars indicate the standard deviations. The differences between the sample with addition of 50 μM DSF and control are statistically significant with *p<0.05, ** p<0.01 as determined by using the Student t test.

**Table S1.** The genes with increased or decreased expression in *B. cereus* after treatment with 50 μM DSF

| **Gene** | | **Accession No.** | **Function** | | | **Fold Change** | | |
| --- | --- | --- | --- | --- | --- | --- | --- | --- |
| BCE_4746 | | AAS43647.1 | Hypothetical protein | | | -24.3 | | |
| BCE_0723 | | AAS39656.1 | Hypothetical protein | | | -17.1 | | |
| BCE_0890 | | AAS39821.1 | periplasmic component of efflux system | | | -10.1 | | |
| BCE_4985 | | AAS43886.1 | ABC transporter | | | -9.5 | | |
| BCE_4107 | | AAS43009.1 | Hypothetical protein | | | -9.3 | | |
| MmgD | | AAS41294.1 | citrate synthase 3 | | | -6.7 | | |
| Sph | | AAS39678.1 | sphingomyelin phosphodiesterase | | | -5.9 | | |
| BCE_3622 | | AAS42527.1 | anaerobic ribonucleoside triphosphate reductase | | | -4.6 | | |
| BCE_3280 | | AAS42188.1 | Hypothetical protein | | | -3.7 | | |
| BCE_4566 | | AAS43467.1 | Hypothetical protein | | | -3.5 | | |
| BCE_2376 | | AAS41294.1 | citrate synthase 3 | | | -3.4 | | |
| BCE_5189 | | AAS44090.1 | Hypothetical protein | | | -3.4 | | |
| BCE_5156 | | AAS44057.1 | chloramphenicol resistance protein | | | -3.3 | | |
| BCE_3792 | | AAS42697.1 | Hypothetical protein | | | -3.2 | | |
| BCE_0797 | | AAS39729.1 | ABC transporter permease | | | -3.2 | | |
| BCE_0225 | | AAS39161.1 | Hypothetical protein | | | -3.2 | | |
| BCE_5195 | | AAS44096.1 | cell surface protein | | | -3.1 | | |
| BCE_0798 | | AAS39730.1 | ABC transporter | | | -3.0 | | |
| BCE_2763 | | AAS41675.1 | Hypothetical protein | | | -3.0 | | |
| BCE_0928 | | AAS39859.1 | Multidrug resistance protein B | | | -2.9 | | |
| BCE_3793 | | AAS42698.1 | 1-phosphatidylinositol phosphodiesterase | | | -2.9 | | |
| BCE_4973 | | AAS43874.1 | Hypothetical protein | | | -2.9 | | |
| BCE_0747 | | AAS39680.1 | flavin-dependent dehydrogenase | | | -2.8 | | |
| BCE_3793 | | AAS42698.1 | 1-phosphatidylinositol phosphodiesterase precursor | | | -2.6 | | |
| BCE_1951 | | AAS40875.1 | chlorohydrolase | | | -2.5 | | |
| BCE_4973 | | AAS43874.1 | Hypothetical protein | | | -2.5 | | |
| BCE_1914 | | AAS40838.1 | 3-oxoacyl-(acyl carrier protein) synthase III | | | -2.5 | | |
| BCE_0817 | | AAS39749.1 | guanine-hypoxanthine permease | | | -2.4 | | |
| BCE_4751 | | AAS43652.1 | Hypothetical protein | | | -2.4 | | |
| BCE_2880 | | AAS41791.1 | Tc resistance protein | | | -2.4 | | |
| BCE_0762 | | AAS39695.1 | amino acid permease family protein | | | -2.4 | | |
| BCE_A0228 | | AAS45076.1 | Hypothetical protein | | | -2.3 | | |
| BCE_0486 | | AAS39421.1 | hydroxyethylthiazole kinase | | | -2.2 | | |
| BCE_0731 | | AAS39664.1 | Multidrug resistance protein B | | | -2.2 | | |
| BCE_0817 | | AAS39749.1 | xanthine/uracil permease family protein | | | -2.2 | | |
| BCE_1296 | | AAS40225.1 | Hypothetical protein | | | -2.1 | | |
| BCE_0800 | | AAS39732.1 | glycine oxidase | | | -2.1 | | |
| BCE_0493 | | AAS39428.1 | ABC transporter | | | -2.1 | | |
| BCE_2620 | | AAS41534.1 | ABC transporter | | | -2.1 | | |
| BCE_0617 | | AAS39551.1 | Hypothetical protein | | | -2.0 | | |
| BCE_4949 | | AAS43850.1 | cytochrome d ubiquinol oxidase subunit | | | -2.0 | | |
| BCE_2927 | | AAS41838.1 | inosine-uridine preferring nucleoside hydrolase | | | -2.0 | | |
| BCE_0617 | | AAS39551.1 | lipoprotein | | | -2.0 | | |
| BCE_4950 | | AAS43851.1 | cytochrome d ubiquinol oxidase, subunit II | | | -2.0 | | |
| BCE_1648 | AAS40577.1 | | Hypothetical protein | | -2.0 | | |  |
| BCE_4558 | AAS43459.1 | | MarR family transcriptional regulator | 2.0 | | |  |  |
| TrpS | AAS40226.1 | | tryptophanyl-tRNA synthetase | 2.0 | | |  |  |
| BCE_2037 | AAS40961.1 | | NLP/P60 family protein | 2.0 | | |  |  |
| BCE_4324 | AAS43225.1 | | sodium:dicarboxylate symporter family protein | 2.1 | | |  |  |
| BCE_3075 | AAS41986.1 | | nitroreductase family protein | 2.1 | | |  |  |
| RpsT | AAS43304.1 | | 30S ribosomal protein S20 | 2.3 | | |  |  |
| BCE_2344 | AAS41262.1 | | thioredoxin family protein | 2.4 | | |  |  |
| BCE_4457 | AAS43358.1 | | Hypothetical protein | 2.7 | | |  |  |
| BCE_3932 | AAS42835.1 | | carbamoyl phosphate synthase small subunit | 2.7 | | |  |  |
| BCE_4764 | AAS43665.1 | | argininosuccinate lyase | 2.8 | | |  |  |
| BCE_4456 | AAS43357.1 | | 5'-methylthioadenosine/S-adenosylhomocysteine nucleosidase | 2.9 | | |  |  |
| RbsA | AAS39668.1 | | ribose ABC transporter ATP-binding protein | 2.9 | | |  |  |
| GntB | AAS41222.1 | | gluconate transporter, permease protein | 3.0 | | |  |  |
| BCE_0736 | AAS3966.1 | | ribose ABC transporter permease | 3.5 | | |  |  |
| BCE_1935 | AAS40859.1 | | acetolactate synthase III, small subunit, truncation | 3.6 | | |  |  |
| BCE_1839 | AAS40764.1 | | lps o-antigen biosynthesis protein | 4.3 | | |  |  |
| BCE_0642 | AAS39575.1 | | malate dehydrogenase, putative | 15.9 | | |  |  |

*Change (fold) in *B. cereus* in the presence of DSF compared to in the absence of DSF determined by microarray analysis.

**A**

**B**

**C**

**D**

**E**

**Figure S2.** The bacterial growth rate of *B. thuringiensis* (A), *S. aureus* (B), *M. smegmatis* (C), *N. subflava* (D), and *P. aeruginosa* (E) in the presence or absence of 50 μM DSF or its analogue. The error bars show the standard deviations of three replicates.

**Figure S3.** Effect of DSF signal and rhamnolipid on the growth rate of *B. thuringiensis*. Bacterial growth was monitored using the Bioscreen-C Automated Growth Curves Analysis System, and the data at 16 h post inoculation were presented here. The error bars show the standard deviations of three replicates.

**Table S2.** Bacterial strains used in this study

| **Strains** | | **Characteristics** | **Growth Temperature (^o^C)** |  | **Source** |
| --- | --- | --- | --- | --- | --- |
| *Bacillus cereus*  ATCC10987 | | Wild- type strain | 37 |  | ATCC |
| *Bacillus*  *thuringiensis* 23 | | *B. thuringiensis* subsp. *israelensis* B23, plasmidless | 37 |  | BGSC |
| *Staphylococcus*  *aureus* | | Mouse isolate | 37 |  | Laboratory collection |
| *Mycobacterium*  *smegmatis* | | Wild- type strain | 28 |  | Laboratory collection |
| *Neisseria subflava* | | Wild- type strain | 37 |  | Laboratory collection |
| *Pseudomonas* *aeruginosa* PA14 | | Clinical isolate | 37 |  | Laboratory collection |
|  |  | |  |  |  |
